# Flexible Lead-Free Piezoelectric Ba_0.94_Sr_0.06_Sn_0.09_Ti_0.91_O_3_/PDMS Composite for Self-Powered Human Motion Monitoring

**DOI:** 10.3390/jfb14010037

**Published:** 2023-01-08

**Authors:** Lin Deng, Weili Deng, Tao Yang, Guo Tian, Long Jin, Hongrui Zhang, Boling Lan, Shenglong Wang, Yong Ao, Bo Wu, Weiqing Yang

**Affiliations:** 1 Key Laboratory of Advanced Technologies of Materials (Ministry of Education), School of Materials Science and Engineering, Southwest Jiaotong University, Chengdu 610031, China; 2 Sichuan Province Key Laboratory of Information Materials, Southwest Minzu University, Chengdu 610041, China

**Keywords:** flexible piezoelectric sensor, BSST particles, piezoelectric composites, BSST/PDMS, human motion monitoring

## Abstract

Piezoelectric wearable electronics, which can sense external pressure, have attracted widespread attention. However, the enhancement of electromechanical coupling performance remains a great challenge. Here, a new solid solution of Ba_1−*x*_Sr*_x_*Sn_0.09_Ti_0.91_O_3_ (*x* = 0.00~0.08) is prepared to explore potential high-performance, lead-free piezoelectric ceramics. The coexistence of the rhombohedral phase, orthorhombic phase and tetragonal phase is determined in a ceramic with *x* = 0.06, showing enhanced electrical performance with a piezoelectric coefficient of *d*_33_~650 pC/N. Furthermore, Ba_0.94_Sr_0.06_Sn_0.09_Ti_0.91_O_3_ (BSST) is co-blended with PDMS to prepare flexible piezoelectric nanogenerators (PENGs) and their performance is explored. The effects of inorganic particle concentration and distribution on the piezoelectric output of the composite are systematically analyzed by experimental tests and computational simulations. As a result, the optimal *V*_OC_ and *I*_SC_ of the PENG (40 wt%) can reach 3.05 V and 44.5 nA, respectively, at 138.89 kPa, and the optimal sensitivity of the device is up to 21.09 mV/kPa. Due to the flexibility of the device, the prepared PENG can be attached to the surface of human skin as a sensor to monitor vital movements of the neck, fingers, elbows, spine, knees and feet of people, thus warning of dangerous behavior or incorrect posture and providing support for sports rehabilitation.

## 1. Introduction

With the rapid development of biomedical and wearable sensors, the monitoring of physiological information related to human healthcare, including respiration [1], heartbeat [2], pulse [3] and behavioral activities [4,5,6], has received widespread attention. So far, many different working principles have been reported for physiological signal monitoring [7,8,9,10,11,12,13,14,15,16,17,18] and good progress has been made. Compared with other existing soft devices based on piezoresistive [9,10,11], capacitive [12], triboelectric [13,14,15,16] or magnetoelastic effects [17], piezoelectric devices have received great interest in the field of flexible wearable electronics due to their simple structure, efficient electromechanical coupling and self-powered characteristics [19,20]. Currently, research on flexible piezoelectric materials has flourished, such as zinc oxide (ZnO) [21,22], poly(vinylidene fluoride) (PVDF) [23,24] and its copolymers [25,26]. However, piezoelectric devices based on such materials are difficult to adopt in high-performance applications due to their relatively low piezoelectric output. On the other hand, piezoelectric ceramics, such as lead zirconate titanate (PZT) [27,28], Pb(Mg_1/3_Nb_2/3_)O_3_-PbTiO_3_ (PMN-PT) [29], potassium sodium niobate (KNN) [30] and bismuth ferrite (BiFeO_3_) [31], exhibit better piezoelectricity, but their inherent brittleness becomes a bottleneck for flexible devices. Accordingly, a more common strategy to develop flexible piezoelectric devices is to prepare inorganic ceramic–organic piezoelectric composites. Nevertheless, PZT and PMN-PT contain toxic elements that threaten human health, while KNN and BiFeO_3_ contain volatile elements that make the materials difficult to prepare and poorly reproducible, further limiting their application in wearable devices.

Barium titanate (BT) [32,33,34] has become one of the most promising piezoelectric materials for the preparation of a piezoelectric nanogenerator (PENG) because of its comparable piezoelectricity with PZT, environmental friendliness and low cost. To obtain flexibility, blending BT particles with polymers is a common strategy. A PENG fabricated by incorporating pure BT particles into an Ecoflex matrix has exhibited some flexibility [35]. In addition, by adding BT particles to a polyvinylidene fluoride–trifluoroethylene copolymer with a kirigami design, the fabricated PENG exhibited some stretchability [36]. However, by blending the piezoelectric inorganic particles into a polymer matrix to form a composite film, the piezoelectric performance was significantly decreased, although the device exhibits some flexibility. This is because the amount of piezoelectric inorganic filler is limited while keeping the thickness and flexibility of the composite film constant. Therefore, it is important to further improve the piezoelectricity of BT-based perovskite ceramics to develop high-performance PENGs for wearable applications.

In this work, we proposed a lead-free flexible PENG based on a composite film consisting of PDMS and BT-based piezoelectric particles with a composition of Ba_0.94_Sr_0.06_Sn_0.09_Ti_0.91_O_3_ (BSST). The BSST ceramics were prepared through the high-temperature solid-phase reaction method. The coexistence of a rhombohedral phase (R), an orthorhombic phase (O) and a tetragonal phase (T) was exhibited near room temperature and it was proven to have an excellent piezoelectric coefficient (*d*_33_~650 pC/N). The BSST particles were used as piezoelectric active substances and the PDMS served as flexible substrates. On this basis, we designed composites and devices with different mass fractions, and their electrical and mechanical performance were investigated by experimental and simulation analysis, respectively. In addition, due to the good flexibility and electrical output of the designed device, conformal contact with human skin can be formed and important human motion signals can be successfully monitored, including of the neck, elbow, spine, knee and foot, which provides the basis for functional applications of flexible wearable electronics.

## 2. Experimental Section

Preparation of the BSST particles: Ba_1−*x*_Sr*_x_*Sn_0.09_Ti_0.91_O_3_ ceramic powder was prepared by the high-temperature solid-phase reaction method using barium carbonate (BaCO_3_, AR 99%), titanium dioxide (TiO_2_, AR 99%), stannic oxide (SnO_2_, AR 99.5%) and strontium carbonate (SrCO_3_, AR 99%). All raw materials were obtained from Shanghai Aladdin Biochemical Technology Co. The raw materials were uniformly mixed with zirconium oxide (ZrO_2_) balls in proportion in ethanol for 24 h. The slurry was dried and then calcined at 1200 °C for 3 h. The calcined powder was mixed with an 8% mass fraction of polyvinyl alcohol (PVA) and pressed into pellets of 13 mm diameter and 0.9 mm thickness under 10 MPa. All samples were kept at 850 °C for 2 h to remove the binder inside the samples. Furthermore, the pellets were sintered in air at 1390 °C for 3 h. In addition, the sintered ceramic sheets were brushed with silver paste as an electrical measurement electrode and calcined at 600 °C for 10 min. Finally, the samples were polarized at room temperature under a direct-current field of 2.5 kV/mm for 25 min.

Fabrication of the PENG: Firstly, a polydimethylsiloxane (PDMS; Sylgard 184, Dow Corning Corporation) solution was prepared by adding a curing agent to the matrix (PDMS in a curing agent ratio of 10:1 by weight). Then, the sintered pellets were then ground with agate for 15 min and finally ball-milled for 12 h to obtain suitable micron-sized powders. The ball-milled BSST ceramic particles were homogeneously mixed with the above-prepared PDMS solution at different concentrations of 3, 5, 10, 20, 30, 40 and 50 wt%. The mixture was spin-coated onto the polyethylene naphthalate (PEN)-indium tin oxide (ITO) flexible substrate at 500 rpm for 18 s and then cured at 80 °C for 3 h. The material was stripped from the flexible substrate and then magnetron sputtering of the silver electrodes was performed on the upper and lower surfaces of the composite. Finally, the composite was polarized under an electric field of 12 kV/mm for 24 h at room temperature.

Characterizations and measurements: The crystal structures of the BSST particles were determined by X-ray diffraction (XRD, DX-1000 diffractometer, 2θ = 20°−60°, PANalytical). The morphologies of BSST particles and composites were observed by field emission scanning electron microscopy (JSM7800F, JEOL). The dielectric constant (ε_r_) of the sample was measured by an LCR analyzer (HP 4980, Agilent, Santa Clara, CA, USA) with varied temperatures between −100~200 °C. The remanent polarization–electric field (*P*–*E*) hysteresis loops and the strain–electric field (*S*–*E*) curves were measured at 1 Hz with a ferroelectric tester (TF Analyzer 2000E, aixACCT). The *d*_33_ of the poled samples was measured by a *d*_33_ meter (ZJ-3A, IACAS) at room temperature. The electrical properties of the devices were tested by Keithley 6514.

## 3. Results and Discussion

**Figure 1:**
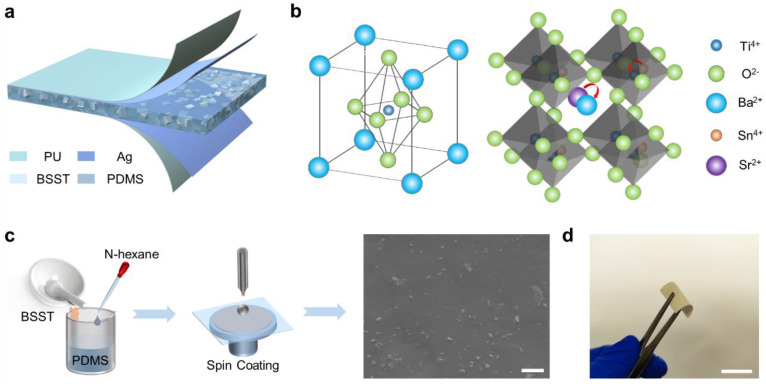
(**a**) Schematic of a flexible sandwich-structured PENG based on BSST/PDMS composites. (**b**) Schematic diagram of the principle of doping Sr and Sn into perovskite-structured BT. (**c**) Preparation process of BSST/PDMS composites. The cross-sectional SEM image of 40 wt% BSST/PDMS composite film. Scale bar, 5 μm. (**d**) Optical photograph of the composite film (2 × 2 cm^2^). Scale bar, 2 cm.

Figure 1a depicts the schematic structure of the sandwich-structured PENG, where two sides of the BSST/PDMS composite are magnetron sputtered with silver as the top and bottom electrodes and then encapsulated with polyurethane tape. As shown in Figure 1b, BT is a perovskite structure, doped with Sr and Sn ions to replace some of the Ba and Ti ions, respectively, to enhance the piezoelectric coefficient. Then, the doped inorganic ceramic particles were mixed with PDMS to fabricate the composite films by the spin-coating method, as shown in Figure 1c. The thickness of the films can be effectively controlled by the rotational speed and spin-coating time (the details of the preparation process can be found in the Experimental Section). From the cross-sectional SEM image of the composite film, it was found that the BSST ceramic particles (40 wt%) were well distributed in the PDMS matrix and the thickness of the composite film was about 323 μm (Figure S1). The optical image in Figure 1d shows a typical flexible BSST/PDMS composite film (40 wt%) with a size of 2 × 2 cm^2^, which can be easily bent to make conformal contact with human skin.

**Figure 2:**
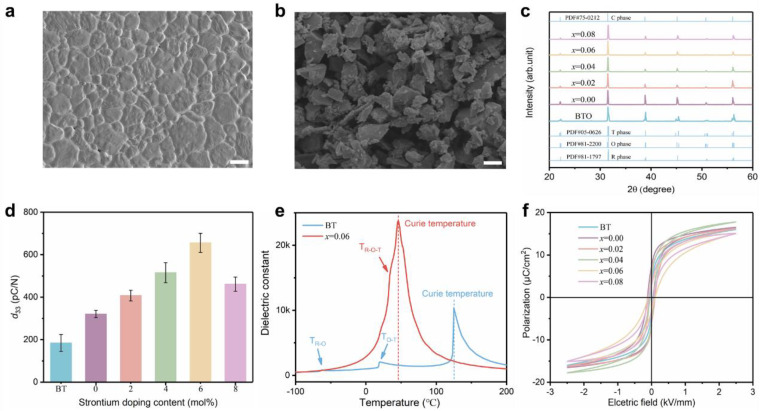
FE-SEM images of BSST block ceramics (**a**) and powder (**b**). The scale bars are 10 μm and 1 μm, respectively. (**c**) XRD patterns of BT and Ba_1−*x*_Sr*_x_*Sn_0.09_Ti_0.91_O_3_ (*x* = 0.00~0.08) ceramics in the 2θ range of 20–60°. (**d**) Piezoelectric coefficient of BT and Ba_1−*x*_Sr*_x_*Sn_0.09_Ti_0.91_O_3_ (*x* = 0.00~0.08) ceramics. (**e**) Temperature dependences of the dielectric constant for the BT and BSST ceramics, measured at -100~200 ℃. (**f**) *P*–*E* hysteresis curve of BT and Ba_1−*x*_Sr*_x_*Sn_0.09_Ti_0.91_O_3_.

In recent years, the construction of multiphase boundaries by chemical modification is the focus of improving the piezoelectric properties of BT-based ceramics. Many studies have demonstrated the feasibility of elemental modification by Ca, Zr, Sn, Hf and Sr [37,38,39,40,41,42,43,44]. Among them, the 9 mol% Sn-doped BT ceramic has a large piezoelectric coefficient, *d*_33_, of 920 pC/N at 50 °C [42]. Additionally, a small amount of Sr-doping can improve the dielectric and ferroelectric properties of BT materials [43]. However, there are fewer reports on the improvement in electrical properties of BT-based ceramics by Sr and Sn co-doping. In this work, we first designed Ba_1−*x*_Sr*_x_*Sn_0.09_Ti_0.91_O_3_ (*x* = 0.00~0.08) lead-free ceramics to obtain a high piezoelectric coefficient by adjusting the phase boundary. All samples showed a dense structure after sintering at 1390 °C (Figure 2a) and then were ground into ceramic powders with a particle size of about 1 μm (Figure 2b). As seen from the X-ray diffraction pattern (Figure S2a), no other diffraction peaks appear before and after grinding the samples, indicating that grinding does not destroy the phase structure of the ceramics. With the doping further increased, all ceramics still show a single perovskite structure without other impurity phases (Figure 2c), indicating that the doped Sr^2+^ and Sn^4+^ completely diffuse into the BT lattice without causing large lattice distortions and forming a stable perovskite solid solution due to the small difference in ionic radii before and after doping. The standard diffraction peaks of R phase (PDF#85-1797), O phase (PDF#81-2200), T phase (PDF#05-0626) and C phase (PDF#75-0212) are indicated by vertical lines. From the magnified X-ray diffraction pattern near 46° (Figure S2b), it can be seen that the diffraction peak of the pure BT ceramic near 46° has two peaks with low left and high right, indicating a T-phase boundary inside the pure BT. After the co-doping of Sr^2+^ and Sn^4+^, the double peaks show signs of fusion, indicating the formation of a multi-phase transition in the component with *x* = 0.00~0.08 at room temperature. As a result, the *d*_33_ of co-doped Ba_1−*x*_Sr*_x_*Sn_0.09_Ti_0.91_O_3_ increases and then decreases with increasing Sr^2+^ content, reaching a maximum at *x* = 0.06 (*d*_33_~650 pC/N), as shown in Figure 2d. To further observe the phase structure of the ceramics, the dielectric properties of BT and Ba_0.94_Sr_0.06_Sn_0.09_Ti_0.91_O_3_ ceramics were measured in the range from −100 to 200 °C at 100 kHz. As shown in Figure 2e, three-phase transition peaks were detected in BT corresponding to the rhombohedral-orthorhombic phase transition temperature (*T*_R-O_), the orthorhombic-tetragonal phase transition temperature (*T*_O-T_) and the cubic phase transition (*T*_C_). For pure BT ceramics, both *T*_R-O_ (~−35 °C) and *T*_O-T_ (~18 °C) are below room temperature, confirming the formation of the T phase, which is consistent with the results of XRD. When the Sr content is 6 mol% and the Sn content is 9 mol%, *T*_R-O_ and *T*_O-T_ gradually approach room temperature, indicating the formation of the R-O-T phase in the sample. Furthermore, the electrical hysteresis loops (*P*–*E*) of BT and Ba_1−*x*_Sr*_x_*Sn_0.09_Ti_0.91_O_3_ ceramics at 1 Hz and 2.5 kV/mm are typical (Figure 2f), indicating that all the samples are ferroelectric. Moreover, the remanent polarization intensity (*P*_r_) gradually decreases with increasing Sr content, as shown in Figure S3. This is mainly due to the distortion of the structure caused by the substitution of Ba^2+^ by Sr^2+^, which has a smaller radius [37], and the lower energy barrier facilitates domain switching and polarization rotation; thus, the irreversible domains of the ceramics are reduced [45,46].

In composites, BSST particles play a key role in generating piezoelectric potential under external stress, while the PDMS matrix provides good flexibility and mechanical sustainability. The operation mechanism of the PENG is schematically illustrated in Figure S4. Before polarization, the dipoles of BSST particles are randomly arranged in the PDMS with zero net dipole moment and no potential difference between the two electrodes, as shown in Figure S4a. After polarization, the disordered dipoles are tilted along the applied electric field, showing a certain order (Figure S4b). In this case, when the device is deformed by the force, the spacing between the top and bottom electrodes changes and the balance of the dipole moment is broken, resulting in an electrical output (Figure S4c). Finally, when the pressure is released, the deformation reverts and there is no electrical output, as shown in Figure S4d.

**Figure 3:**
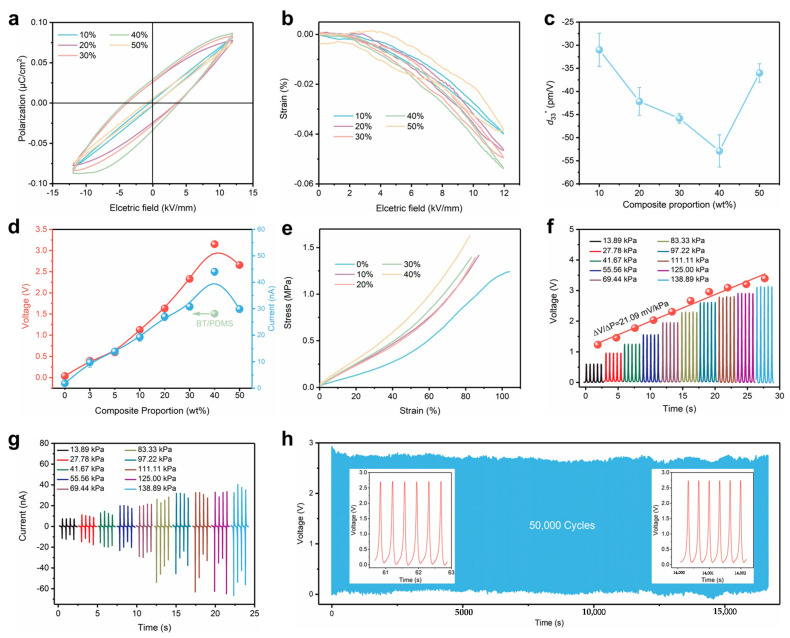
(**a**) *P*–*E* hysteresis curves of the composite film at different BSST concentrations. (**b**) Strain curves of composite films under different electric fields. (**c**) Piezoelectric coefficient (*d*_33_) as a function of BSST concentration. (**d**) Open-circuit voltage and short-circuit current of the PENG as a function of BSST concentration. (**e**) Stress–strain curves of composite films. Output results of open-circuit voltage (**f**) and short-circuit current (**g**) under different pressures with the BSST concentration of 40 wt%. (**h**) Durability tests were conducted to confirm the mechanical stability of the device.

From the ferroelectric hysteresis of the BSST/PDMS composites, it can be seen that the remanent polarization intensity (*P*_r_) tends to increase and then decrease as the Ba_1−*x*_Sr*_x_*Sn_0.09_Ti_0.91_O_3_ particle concentration increases from 10 to 50 wt% (Figure 3a). It can be inferred that the disordered electric dipoles in the composites tend to align with the polarization of the external electric field [47]. The composite with 40 wt% exhibits the best performance with a corresponding *P*_r_ and coercivity electric field (*E*_c_) of 0.03 μC/cm^2^ and 4.01 kV/mm, respectively. To understand the piezoelectricity of the composite, the *S*–*E* curves of the composites were also tested and are shown in Figure 3b, based on the inverse piezoelectric constant ($d_{33}^{*}$) calculated as:

$$d_{33}^{*}=\frac{dS}{dE}$$

where *S* is the strain of the material under the action of the applied electric field, *E*. The strain of the material shows a similar trend, which is that the strain tends to increase and then decrease with the increase in BSST particle concentration, with the piezoelectric coefficient reaching a maximum of ~−53 pm/V at 40 wt%, as seen in Figure 3c. In composites, the piezoelectric output mainly originates from ceramic fillers after polarization. When polarizing the composite, excessive ceramic particle aggregation tends to cause charge accumulation and can lead to breakdown due to the large difference in dielectric constants between the ceramic fillers and the matrix. Therefore, a series of PENGs (effective area: 1.2 × 1.2 cm^2^) with different mass fractions were prepared to investigate the effect of inorganic particle concentration on the output performance of PENG. As can be seen from Figure 3d, the output voltage and current of the PENG gradually increased with the increase in BSST particle concentration, reaching a maximum of 3.05 V and 44.5 nA for the PENG with a concentration of 40 wt% at 138.89 kPa, which is approximately a two-fold increase compared to the BT/PDMS composite. However, when the BSST particle concentration was further increased to 50 wt%, the output weakened. The response of the composite to each concentration at different pressures is shown in Figure S5. The results suggest that the concentration of BSST particles in the composites has a significant effect on the piezoelectric output of the PENG, and that there is an optimal mass ratio. It can be seen that the number of particles in the cross-sectional SEM images gradually increased, and an obvious agglomeration was observed in the 50 wt% composite films (Figure S6a,b), which is due to the significant difference in the surface energy between the ceramic filler and the polymer matrix. When the content of inorganic filler increases, the inorganic particles tend to agglomerate in the polymer matrix to form a more stable low-energy state [48], which will easily cause a breakdown, resulting in a decrease in the output performance of PENG. On the other hand, according to the piezoelectric equation, the model can be simplified to [49,50]:

$$d_{33}=\frac{\partial P_{3}}{\partial \sigma_{3}}\approx -\frac{P_{r}}{Y}$$

where $P_{3}$ is the intensity of polarization, $\sigma_{3}$ is the uniaxial stress, *P*_r_ is the remanent polarization strength and *Y* is the Young’s modulus. This equation uses the deformation of amorphous regions to describe the piezoelectric effect in polymers and is the traditional dimensional model for describing the *d*_33_ of piezoelectric polymers. Perovskite ceramics are stiffer (higher modulus of elasticity) than polymer materials, and as a result, the dimensional effect of the polymer is much stronger. Thus, the model can explain two-thirds of the piezoelectric activity, with the rest of the contribution coming from the dipole moment in the crystal region [51]. In this work, the addition of inorganic particles increases the Young’s modulus of the material, leading to a decrease in strain under the same external force, which was confirmed in stress–strain tests of the composite shown in Figure 3e and Figure S7a. In addition, the remanent polarization strength of the material increases, which in turn favors the piezoelectric output. Additionally, when the number of BSST particles is further increased to 50 wt%, the remanent polarization strength decreases sharply and the output signal weakens. To confirm that the PENG can be used to capture and recognize different movements of the human body, the open-circuit voltage (Figure 3f) and short-circuit current (Figure 3g) output of a 40 wt% PENG were tested under different pressures, and the voltage sensitivity reached 21.09 mV/kPa. In addition, the output voltage of the device did not change significantly during 50,000 consecutive compression shocks, verifying the good durability of the PENG, as shown in Figure 3h.

**Figure 4:**
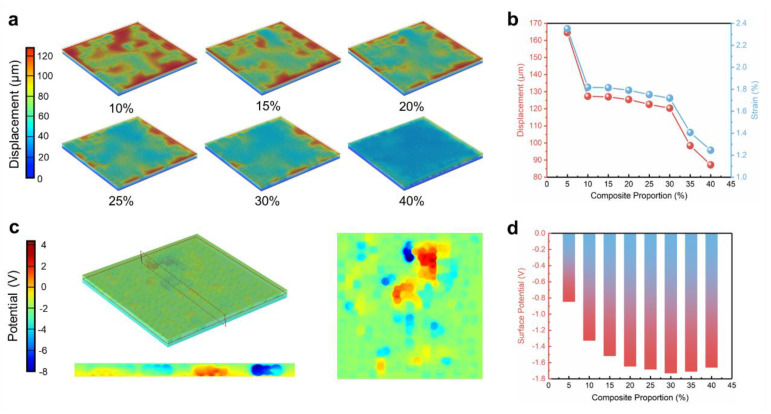
The simulation results of the PENG. (**a**) Deformation of the BSST/PDMS composite with different volume fractions under 200 kPa pressure. (**b**) Statistical results of maximum displacement and strain of composite films with different volume fractions under 200 kPa pressure. Potential distributions in transverse and longitudinal sections (**c**) and surfaces (**d**) under 200 kPa pressure for a 30% volume fraction of the composite.

Furthermore, finite element analysis was used to further verify and illustrate the effect of the Young’s modulus and the distribution of particles on the macroscopic piezoelectricity of the material using COMSOL software. To facilitate the calculations, the bottom surface of the piezoelectric composite was defined as the zero potential plane in the simplified model and a vertical compression force was applied to the upper surface. The simulation results show that the deformation of the material decreases with the increase in the composite fraction (Figure 4a) and increases with the pressure (Figures S7b and S8a). The deformation is greater when the inorganic particles are uniformly dispersed compared to when they are aggregated, and the statistics of the maximum displacement and strain in the composite films with different volume fractions at 200 kPa are shown in Figure 4b. It means that the introduction of inorganic ceramic particles increased the modulus of elasticity of the material, resulting in a reduction in strain under the same external force. The cross-sectional and longitudinal potential distribution of the composite with a 30% volume fraction at a pressure of 200 kPa is displayed in Figure 4c, and surface potentials of the composites with other fractions can be found in Figure S8b. The results show that there is a clear difference in the potential along different directions due to the different distribution of inorganic fillers. From the simulation results of the surface potential of composites with different volume fractions (Figure 4d), it can be seen that the surface potential tends to increase and then decrease with the increase in volume fraction, with the overall trend in agreement with the experimental results.

**Figure 5:**
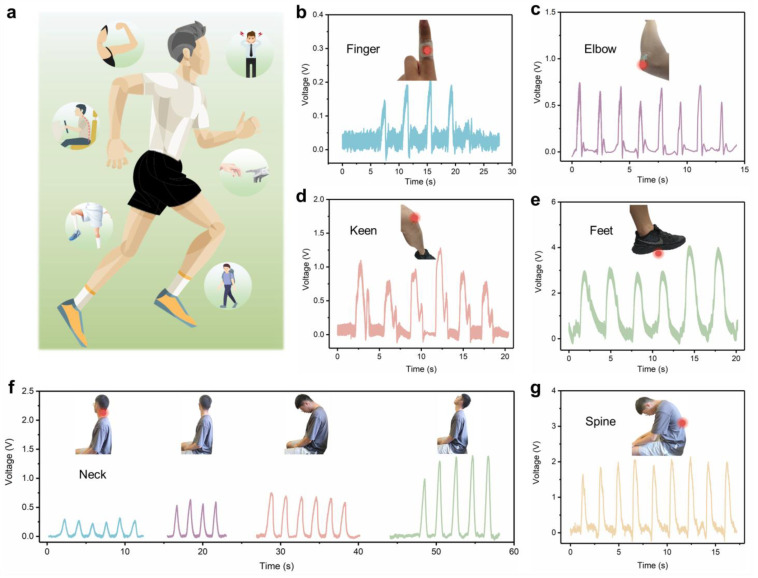
(**a**) Schematic diagram of a PENG based on a BSST/PDMS composite used for human motion monitoring. Measurement of human movements: fingers (**b**), elbows (**c**), knees (**d**), feet (**e**), neck (**f**) and spine (**g**).

Due to the excellent flexibility and electrical properties discussed above, the as-prepared self-powered PENG based on BSST/PDMS has been successfully used for human motion monitoring (Figure 5a). Without any external power supply, it was used as a self-powered wearable biomechanical sensor for different application scenarios. When the device was attached to the finger, the finger flexion could cause the device to produce a stable voltage of about 0.2 V, which can be used for finger motion monitoring (Figure 5b). Similarly, when the device was attached to the elbow, knee and foot, voltages of about 0.65 V, 1 V and 3.5 V, respectively, could be detected, as shown in Figure 5c–e. Thus, the device can be used to simply identify different limb movements of the human body. In addition, BSST/PDMS piezoelectric sensors could also be installed on the neck and spine to monitor their movements. When the head of the tester moved from side to side or back and forth at different distances, the device generated output voltages of different amplitudes that could be used for the detection of driving with fatigue by monitoring human head movements (Figure 5f). The bending motion of the human spine caused a significant piezoelectric signal from the attached device, providing potential for human spine posture monitoring (Figure 5g). In the future, this sensor may also be used to remind sedentary people to exercise as well as for human rehabilitation training and monitoring of intelligent robot movements.

## 4. Conclusions

In summary, we successfully prepared a high-performance lead-free flexible PENG based on BSST by a high-temperature solid-phase reaction and spin-coating method, and demonstrated its application as a flexible self-powered piezoelectric sensor. It was demonstrated that Sr and Sn co-doping can modify the *T*_R-O_ and *T*_O-T_ to room temperature, eventually forming a multiphase boundary of R-O-T, resulting in enhanced piezoelectric properties (190 pC/N to 650 pC/N). After blending with PDMS, the piezoelectricity of the composite shows a trend of increasing and then decreasing, and an optimal mass ratio exists. The open-circuit voltage and the short-circuit current of the device with a concentration of 40 wt% can reach 3.05 V and 44.5 nA at 138.89 kPa, respectively. Moreover, the monitoring of human joint motions was successfully demonstrated by the prepared PENG, which lays the foundation for sports monitoring. This work paves a new route for the development of high-performance human motion rehabilitation monitoring as well as flexible human–machine interactions.

## Data Availability

The data presented in this study are available on request from the corresponding author.

## Supplementary Materials

The following supporting information can be downloaded at: https://www.mdpi.com/article/10.3390/jfb14010037/s1, Figure S1: Cross-sectional SEM image of the composites with 40 wt%; Figure S2: X-ray diffraction of ceramics of different components. Figure S3: The remanent polarization intensity (*P*_r_) of the samples with different content of Sr. Figure S4: The operation mechanism of the PENG. Figure S5: Piezoelectric output of PENG. Figure S6: The cross-sectional SEM image of 30 wt% and 50 wt% composite films. Figure S7: Young’s modulus of the composites and simulation results. Figure S8: Schematic diagram of simulation results.
