# Supplementary material for: Flexible Lead-Free Piezoelectric Ba0.94Sr0.06Sn0.09Ti0.91O3/PDMS Composite for Self-Powered Human Motion Monitoring
Source: J Funct Biomater. 2023 Jan 8;14(1):37. doi: 10.3390/jfb14010037 (PMC9860964; doi:10.3390/jfb14010037)
Supplement: Supplementary file 1 [file jfb-14-00037-s001.zip › jfb-2096564-supplementary.pdf]

Flexible Lead-Free Piezoelectric $Ba_{0.94}Sr_{0.06}Sn_{0.09}Ti_{0.91}O_3$/PDMS Composite for Self-Powered Human Motion Monitoring

Lin Deng[1], Weili Deng[1], Tao Yang[1], Guo Tian[1], Long Jin[1], Hongrui Zhang[1], Boling Lan[1], Shenglong Wang[1], Yong Ao[1], Bo Wu[2]* and Weiqing Yang[1]*

[1] Key Laboratory of Advanced Technologies of Materials (Ministry of Education), School of Materials Science and Engineering, Southwest Jiaotong University, Chengdu 610031, China

Prof. W. Deng

* Correspondence: weili1812@swjtu.edu.cn

Prof. B. Wu

[2] Sichuan Province Key Laboratory of Information Materials, Southwest Minzu University, Chengdu 610041, China

* Correspondence: wubo7788@126.com

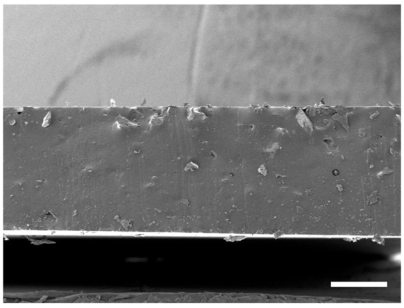

Figure S1. Cross-sectional SEM image of the composites with 40 wt%. Scale bar, 100 μm.

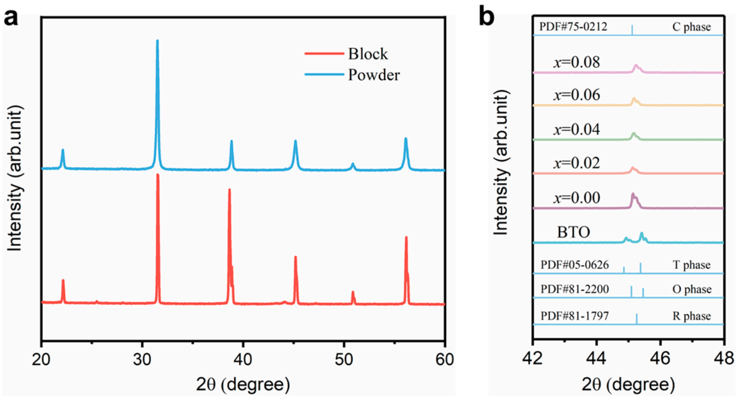

Figure S2. X-ray diffraction of ceramics of different components. (a) X-ray diffraction patterns of block and powder ceramics at $x = 0.06$ in the 2θ range of 20-60°. (b) X-ray diffraction patterns of the BT and $Ba_{1-x}Sr_xSn_{0.09}Ti_{0.91}O_3$ ceramics in the 2θ range of 42-48°.

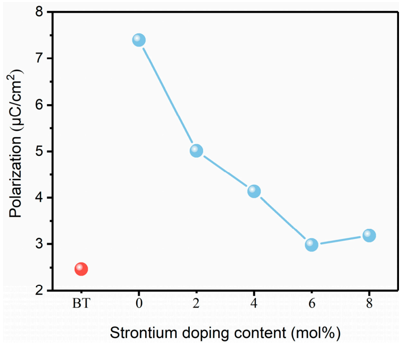

Figure S3. The remanent polarization intensity (P_r) of the samples with different content of Sr.

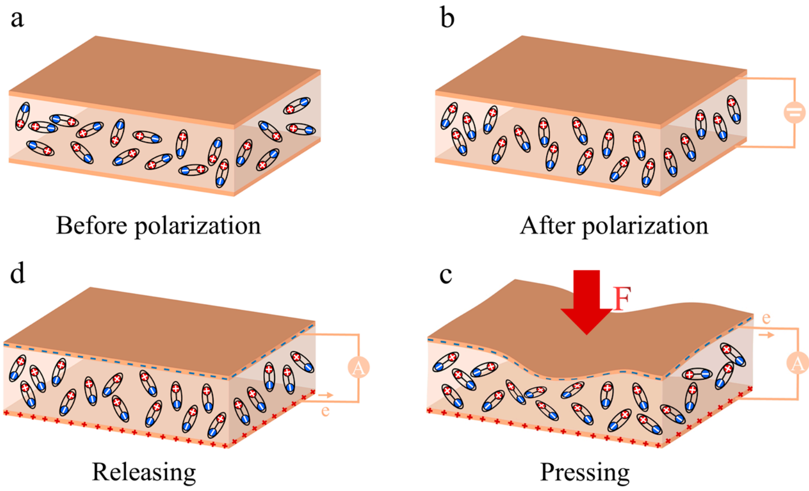

Figure S4. The operation mechanism of the PENG. (a) The dipoles in the composite are randomly aligned before polarization. (b) After polarization, the dipoles inside the composite will be aligned along the electric field. (c)When pressure is applied to the composite, the balance of the dipole moment is broken and an electric potential difference is generated between the upper and lower electrodes. (d) When the pressure is released, the accumulated electrons flow back in the opposite direction.

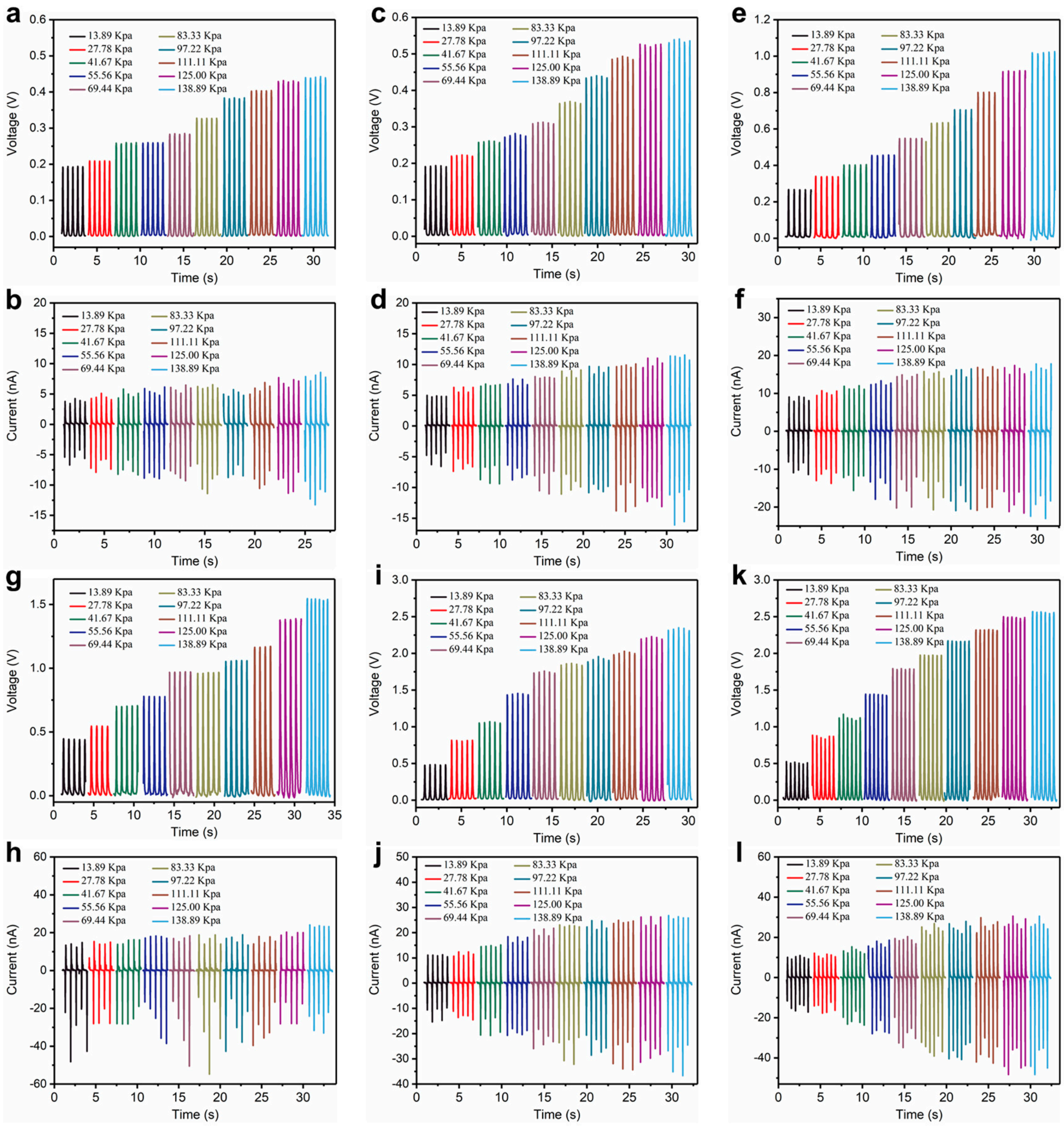

Figure S5. Piezoelectric output of PENG. (a)-(l) Open-circuit voltage and short-circuit current of PENG with different mass fractions under different pressure conditions (3, 5, 10, 20, 30, 50 wt%).

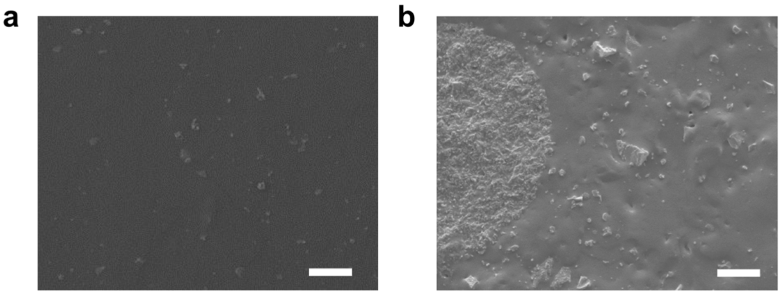

Figure S6. The cross-sectional SEM image of 30 wt% (a) and 50 wt% (b)composites films. Scale bar, 5 μm.

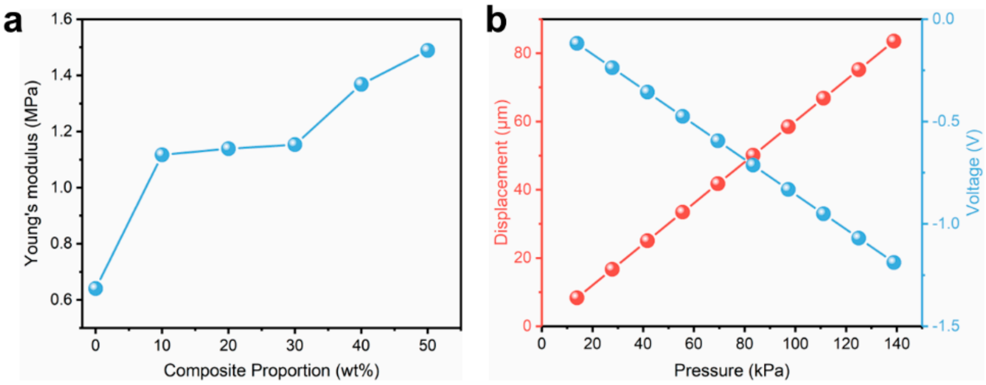

Figure S7. Young's modulus of the composites and simulation results. (a) Young's modulus of composites with different mass fractions. (b) Simulation of deformation and terminal voltage of 30% volume fractions composites under different pressures.

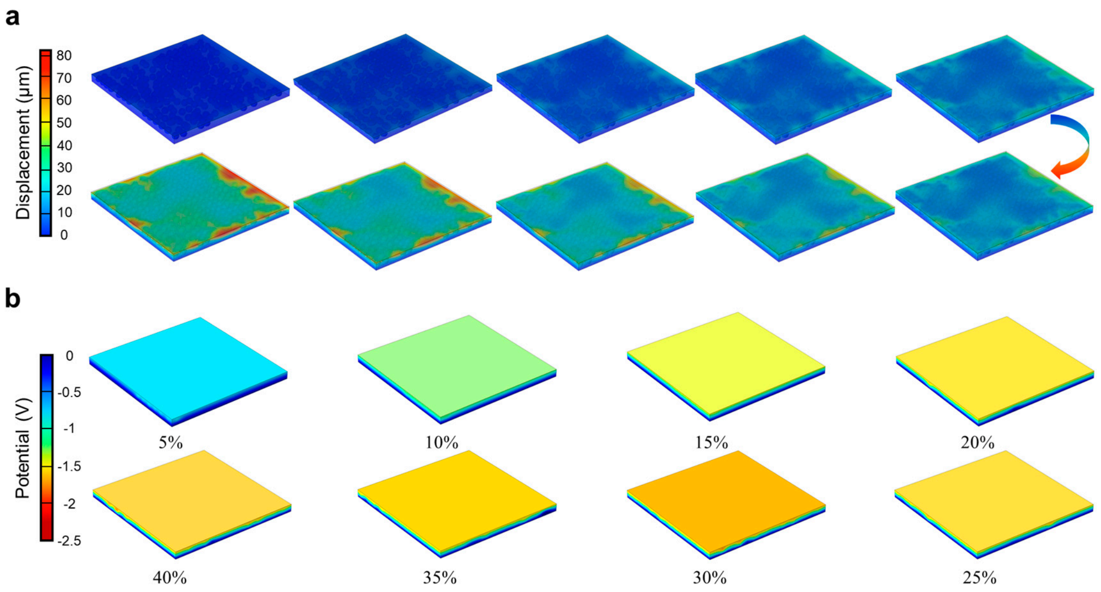

Figure S8. Schematic diagram of simulation results. (a) The deformation of 30% volume fraction composites under different pressures, ranging from 13.89 kPa-138.89 kPa. (b) Simulation of surface potential for composites with different volume fractions at 200 kPa pressure.
